# Synthesis, Characterization, and Biological Studies of Biopolyurethane-Chitosan Composites Based on Diphenylmethane Diisocyanate and Polyol Derived from Castor Oil for the Development of Biomaterials for Topical Use

**DOI:** 10.1021/acsomega.5c02165

**Published:** 2025-05-22

**Authors:** Ricardo dos Santos Medeiros, Ana Paula Garcia Ferreira, Carolina K. Sanz, Sara Gemini Piperni, Kaio Pini Santos, Marlus Chorilli, Wagner Luiz Polito, Tiago Venâncio, Éder Tadeu Gomes Cavalheiro

**Affiliations:** † Instituto de Química de São Carlos, 153988 Universidade de São Paulo, Av. Trabalhador São-carlense, 400, 13566-590 São Carlos, SP, Brazil; ‡ Laboratório de Biotecnologia, Bioengenharia e Biomtariais Nanoestruturados (LabeN), Instituto de Ciências Biomédicas, 28125 Universidade Federal do Rio de Janeiro, Campus Cidade Universitária, 21941 Rio de Janeiro, RJ, Brazil; § School of Pharmacuetical Sciences − Rodovia Araraquara Jaú, São Paulo State University, Km 01 − s/n−Campos Ville, 14800-903 Araraquara, SP, Brazil; ∥ Departamento de Química- DQ, 67828 Universidade Federal de São Carlos, Rodovia Washington Luis s/n Km 235, 13565-905 São Carlos, SP, Brazil

## Abstract

Polyurethanes (PUs) are polymers that have aroused considerable interest in the medical and tissue engineering fields due to their physicochemical properties, such as mechanical and thermal stability, elasticity, and biocompatibility. PUs have been used in the manufacturing of medical devices since the 1960s, such as catheters, artificial hearts, and blood bags. This class of polymers is synthesized from the polyaddition reaction of a polyol (soft segment) with a diisocyanate (hard segment). In view of the tendency to replace polyols derived from fossil resources with those derived from renewable resources, vegetable oils and byproducts of hydroxylated biomass are seen as emerging raw materials for the synthesis of PUs. Raw materials derived from renewable sources include polysaccharides (starch, cellulose, and chitosan) as well as fats and oils of a vegetable and animal origin and have been employed in the creation of biomaterials. Castor oil extracted from the seeds of the plant Ricinus communis is a natural polyol made up of 89% of ricinoleic acid, with an 18-carbon chain and two groups subject to reaction: an unsaturation on carbon 9 and a hydroxyl on carbon 12. Chitosan (CTS) is a polysaccharide in the form of a copolymer formed from 2-amino-2-deoxy-d-glucopyranose and 2-acetamido-2-deoxy-d-glucopyranose units randomly linked by β (1 → 4) glycosidic bonds. Its physicochemical and biological properties make CTS attractive for various applications due to its biocompatibility, biodegradability, mucoadhesivity, and absence of toxicity, along with antimicrobial activity, the ability to coordinate metals, and the ability to serve as a matrix for the loading and controlled release of substances. In the present study, biopolyurethane-chitosan (PUCTS) composites were prepared using the “one-shot method”, in which a polyol derived from castor oil was mixed with CTS and then with methylene diphenyl diisocyanate (MDI) in a reaction flask at room temperature with constant stirring. The mixture was degassed and poured into a silicone mold for curing at room temperature. The polymers were characterized by FTIR, ^13^C NMR, X-ray diffraction (XRD), scanning electron microscopy (SEM), and thermoanalytical methods (thermogravimetric analysis (TGA) and dynamic mechanical analysis (DMA)), followed by the investigation of cytotoxicity and cell adhesion on the surface of the composite. The hydroxyl number determined for the polyol was 304 mgKOH/g, and the isocyanate content was 36%. FTIR spectroscopy revealed changes in the profiles of the OH bands of the polyol in the 3500–3200 cm^–1^ region, the disappearance of the N=C=O band of MDI at 2189 cm^–1^ and the increased intensity of the C=O and N–H bands in the 1750–1500 cm^–1^ region due to the formation of the urethane bond. ^13^C NMR demonstrated the presence of CTS in the PU matrix, and the XRD graphs illustrated the amorphous, crystalline region of the polymers. SEM revealed roughness on the surface and inside the PU as well as circular spots with diameters smaller than 200 μm, which are characteristic of the outflow of gases during polymerization. The TGA curves of PUCTS showed the loss of mass, with thermal stability ranging from around 170–200 °C. Based on the DMA curves, the glass transition was between 16 and 20 °C. The biological test revealed that PUCTS exhibited mild cytotoxicity, and cell adhesion tests revealed that PU90CTS10 and PU50CTS50 composites promoted cell adhesion in the fibroblast cell line (L929). The results demonstrated the potential of the PUCTS composite for application as a biomaterial for topical use (bandage), with the ability to insert drugs to accelerate the healing process.

## Graphical abstract

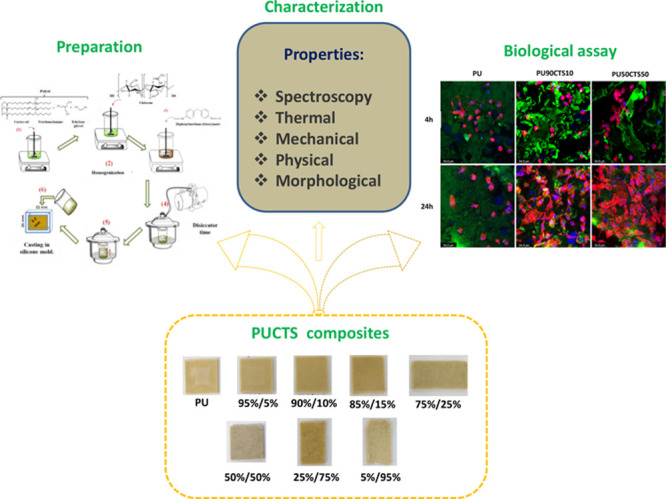

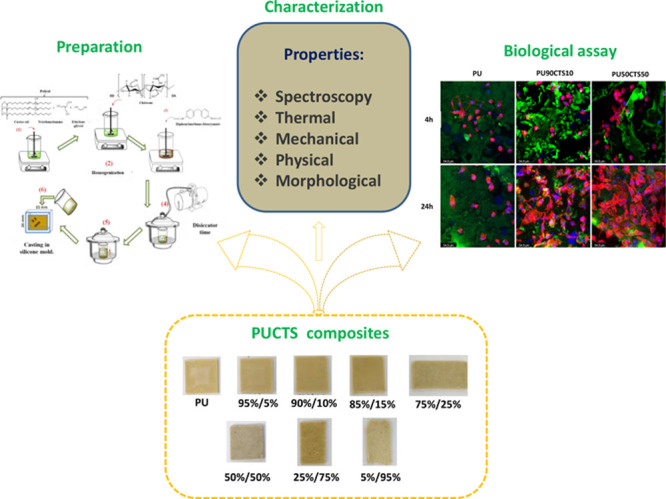

## 1 Introduction

The European Society of Biomaterials defines a biomaterial as a “material intended to interact with biological systems to evaluate, treat, augment, or replace any tissue, organ, or function of the body”. The Journal of Biomaterials defines a biomaterial as “a substance that has been designed to assume a form that, alone or as part of a complex system, is used to direct, by controlling interactions with components of living systems, the course of any therapeutic or diagnostic procedure”.^1^

The development of science and technology has led to the creation of novel biomaterials designed for specific applications. The functions of these materials are governed by their physicochemical, mechanical, and biological properties. The production of biomaterials occurs in an interdisciplinary way, involving different fields, such as medicine, biology, physics, chemistry, tissue engineering, and materials science.^2^

Polymeric biomaterials are among the most widely used materials in the biomedical field, offering advantages in terms of cost compared with materials of a ceramic or metallic origin. Thus, there is a range of polymers of synthetic or natural origin for the creation of biomaterials that can be used in various applications. Biomaterials produced from synthetic polymers include nylon, poly­(vinyl alcohol) (PVA), polyethylene terephthalate (PET), polycrapolactone (PCL), polylactic acid, polypropylene, silicone rubber, and polyurethane.^3^

Biopolymers derived from living matter (proteins, nucleic acids, and polysaccharides), such as chitosan, alginate, collagen, and gelatin, can be used in the creation of biomaterials. Moreover, material composites, in which these polymeric matrices are reinforced with inorganic particles, such as alumina, hydroxyapatite, bioglass, silica, and titanium, are also employed in the creation of biomaterials.^3,4^

Polyurethanes (PUs) are polymers that have aroused considerable interest in the medical and tissue engineering field due to their physicochemical properties, such as mechanical and thermal stability, elasticity, and biocompatibility.^5^ PUs can be inert or have specific objectives, offering characteristics such as absorptivity, biodegradability, and regenerativity.^5,6^ According to Guelcher and collaborators, PUs have been used in the manufacturing of medical devices since the 1960s, such as catheters, artificial hearts, and blood bags.^7^ Furthermore, there are reports of applications in the regeneration of bone tissue, with the implantation of PUs in bone defects, promoting greater blood supply and cell growth.^6,8^

PUs are synthesized from the polyaddition reaction of a polyol (di- or more functional R–OH) with an isocyanate (di- or trifunctional R–N=C=O) in the presence or absence of additives and catalysts. PUs are polymers whose polymer chain has units of the carbamate bond (−NH–CO–O−), which in polymer chemistry is known as the urethane bond. Polyalcohols of a fossil, synthetic, or vegetable origin as well as aromatic and aliphatic diisocynates can be used in the preparation of PUs.^9–11^ This route is known as classic PU synthesis, which has three methods: “one shot”, prepolymer, and quasi-prepolymer.^11^ Figure 1 displays the PU formation reaction.

In view of the tendency to replace polyols derived from fossil resources with renewable resources, vegetable oils and byproducts of hydroxylated biomass are seen as emerging raw materials for the synthesis of PUs.^12–14^ In addition to their environmental appeal, these raw materials have different properties compared to those of PUs of a fossil origin. Raw materials derived from renewable sources include polysaccharides (e.g., starch, cellulose, and chitosan) as well as fats and oils of a vegetable and animal origin and have been employed in the creation of biomaterials.^15,16^

Vegetable oils are viscous liquids made up of triglyceride molecules (fatty polyesters) extracted from plant seeds, such as soybean, sunflower, canola, linseed, castor bean, etc. Castor oil extracted from the seeds of the plant Ricinus communis is a natural polyol made up of 89% ricinoleic acid, with an 18-carbon chain and two groups subject to reaction: an unsaturation on carbon 9 and a hydroxyl on carbon 12, and 11% other fatty acids.^15,16^ The literature offers studies on PUs derived from vegetable oils, such as castor oil, with characteristics ranging from rigid, semirigid or flexible foams to elastomers and composites with other polymers.^17–29^

Chitosan (CTS) is a polysaccharide in the form of a copolymer formed from 2-amino-2-deoxy-d-glucopyranose and 2-acetamido-2-deoxy-d-glucopyranose units randomly linked by β (1 → 4) glycosidic bonds, Figure 2.^30,31^ CTS can be obtained by the alkaline hydrolysis of chitin in a reaction known as deacetylation, which results in a reaction product with an average degree of deacetylation of ≥50%.^31,32^

Its physicochemical and biological properties make CTS attractive for various applications due to its biocompatibility, biodegradability, muco-adhesivity, and absence of toxicity, along with antimicrobial activity, the ability to coordinate metals, and the ability to serve as a matrix for the loading and controlled release of pharmaceuticals.^33,34^ Many of these properties depend on the acetylation pattern.

CTS can be used in composites with other materials, resulting in films, membranes, nanofibers, nanoparticles, etc., and has gained prominence in several fields, such as tissue engineering, wound dressing, and pharmaceuticals, as it enhances the properties of materials by promoting a synergistic effect.^35^ The literature offers several studies that have derived biomaterials from chitosan and PUs obtained from polyols of vegetable or synthetic origin. Such materials are prepared as nanofibers,^36–39^ films,^40–44^ hydrogels,^45^ membranes,^46^ and drug delivery systems.^47^

Due to the characteristics of these polymers demonstrated in the studies cited above, the aim of the present study was to prepare polyurethane/chitosan (PUCTS) composites using castor oil as a polyol (renewable material), with the investigation of its properties and biological activity when tested in vitro for application as a biomaterial to be used in the dressing of wounds.

## 2 Materials and Methods

Diphenylmethane diisocyanate (MDI) (Domain Tecnologia Qumica, Ltd.) and a polyol composed of castor oil, triethanolamine, and ethylene glycol were used to prepare the polyurethane of a vegetable origin. Low molar mass chitosan (*M̅*_v_ = 43000.0 ± SD = 4450.00 Da) (Sigma-Aldrich) was used in the preparation of the PUCTS composite. Chitosan was purified as described in the next section.

### 2.1 Chitosan Purification

Chitosan was purified using a procedure adapted from Signini and collaborators,^48,49^ in which 5.0 g of commercial chitosan was added to a 3% acetic acid solution (v/v) and kept under stirring for 18 h. The mixture was then filtered through a sintered glass funnel. To precipitate the chitosan, 100 mL of concentrated ammonium hydroxide (NH_4_OH) was added, followed by stirring for 1 h. The precipitate was washed with water until reaching pH between 7 and 8. The material was then filtered and washed with ethanol. The sample was dried under low pressure in a vacuum oven at 40 °C for 72 h. After being dried, the material was crushed manually with an agate pestle and mortar, resulting in a yellowish powder.

**Figure 1:**
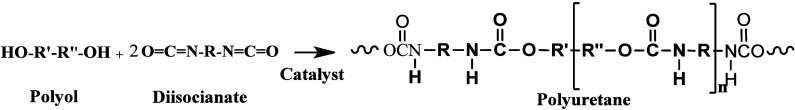
Representation of the reaction of PU synthesis.

**Figure 2:**
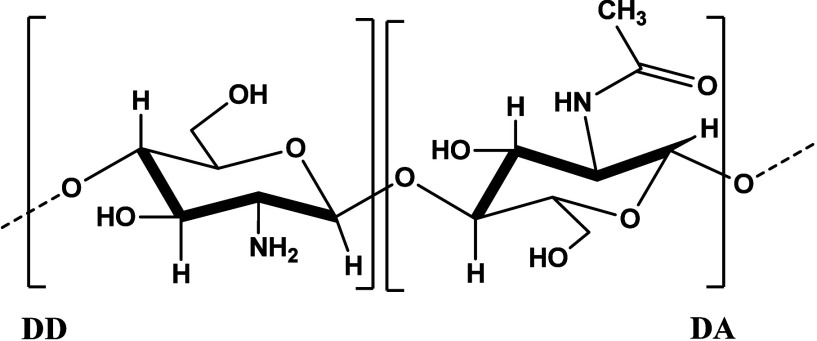
Structural representation of polymer chain units in chitosan.

### 2.2 Polyurethane Preparation

The polyol prepared from mixtures of polyalcohol formed by castor oil, triethanolamine, and ethylene glycol in a 3:3:1 molar proportion of castor oil, triethanolamine, and ethylene glycol, respectively, was treated with the passage of N_2_ for 6 h in a closed system with mechanical stirring at 50 °C in a three-neck round-bottom flask

To prepare the polymers, 5.0 g of the polyol was weighed directly in the reaction flask, followed by the addition of 3.75 g of MDI. The mixture was stirred for 5 min and placed in a desiccator with a vacuum pump for a period of 3 min to remove the bubbles resulting from the elimination of CO_2_. The sample was then cast in a silicone mold to cure the material at room temperature.^6^

### 2.3 Preparation of the Polyurethane/Chitosan Composite (PUCTS)

When preparing the composite, the mass ratio between PU and CTS varied, with 0, 5, 10, 15, 25, 50, 75 and 95% chitosan (m/m). Table 1 displays the proportions used to prepare the composites. A schematic of the composite preparation procedure is shown in Figure 3.

**Figure 3:**
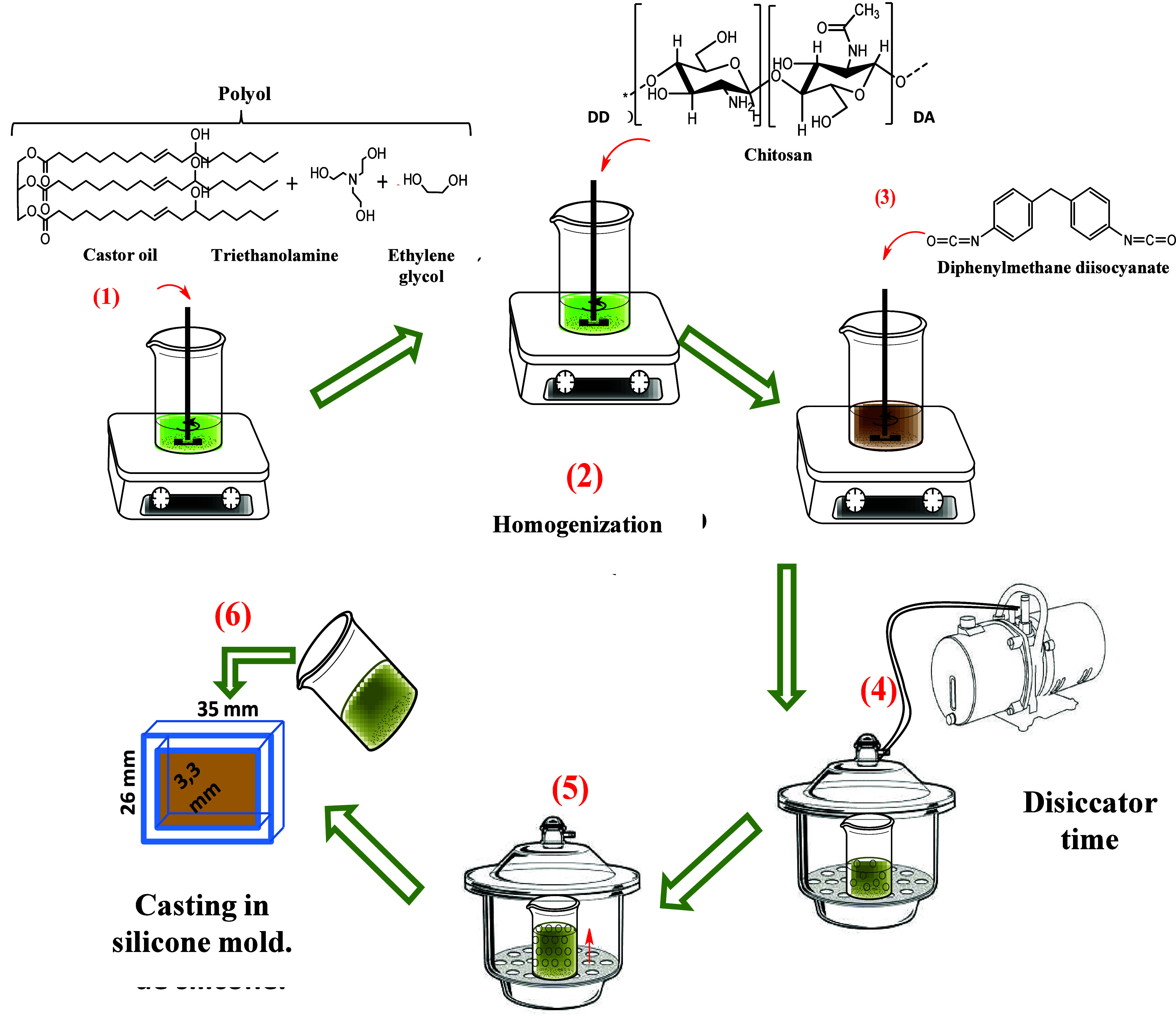
Scheme represents the preparation of PU and PUCTS composites.

**Table 1:** Proportion Used between Reagents for Preparation of PUCTS Composites

sample	polyola (g)^a^	MDIb (g)^b^	CTSc (g)^c^
PU	5	3.75	0
PU95CTS5	4.75	3.56	0.25
PU90CTS10	4.5	3.37	0.5
PU85CTS15	4.25	3.18	0.75
PU75CTS25	3.75	2.81	1.25
PU50CTS50	2.51	1.88	2.49
PU25CTS75	0.63	0.46	1.87
PU5CTS95	0.13	0.09	2.37

a Mass value calculated based on the hydroxyl index.

b Mass value obtained from % NCO free.

c Mass value calculated from of PU pure, multiplying the percentage required. Numbers represent the % of each component.

The procedure is similar to the previous one. Initially, the desired mass of chitosan was dispersed in the previously weighed polyol. The mixture was stirred for 5 min until homogeneous and placed in a desiccator for 10 min to remove air bubbles. The desired mass of MDI was then added and the mixture was stirred for another 5 min, after which the system was placed back in the desiccator for 3 min.^9^ Lastly, the sample was cast in a silicone mold.

### 2.4 Characterization of PU and PUCTS Composites

#### 2.4.1 Solid-State Carbon Nuclear Magnetic Resonance Spectroscopy (^13^C NMR)

Solid-state ^13^C NMR spectra were obtained in a Bruker Avance III-400 spectrometer–9.4 T (399.94 MHz for the hydrogen nucleus) equipped with a 4 mm magic angle spinning solid-state probe. Samples were placed in a 4 mm rotor, which was rotated around its magic angle at 5 kHz.

#### 2.4.2 Fourier Transform Infrared Spectroscopy (FTIR)

Vibrational spectra in the infrared region were obtained with an IRAffinity-1 FTIR spectrophotometer (Shimadzu) in the 400–4000 cm^–1^ range with a resolution of 4 cm^–1^ and 32 acquisition scans. Samples were prepared as tablets containing a mixture of 5.0 mg of sample to 95.0 mg of potassium bromide (KBr) previously dried in an oven at 90 °C. During the preparation of the tablets, both the samples and KBr salt were kept under a 100 W incandescent lamp to prevent moisture absorption.

#### 2.4.3 X-ray Diffraction (XRD)

XRD analyses of the polyurethane and composite samples were carried out by using a Rigaku powder diffractometer (Ultima IV model) equipped with copper as a radiation source in normal scanning mode. Data were acquired in 2θ/θ mode, with a scan from 3° to 80°, step of 0.02 s^–1^, and velocity of 0.5 min^–1^. The crystallinity and amorphicity indices of the PU samples and composites were determined using the method proposed by Cassales (2020) and Alexander (1969).^23,50^

#### 2.4.4 Thermal Analysis

##### 2.4.4.1 Thermogravimetric Analysis (TGA)

The thermogravimetric and differential thermal analysis curves of the purified chitosan samples, polyurethane, and composites were obtained using the simultaneous TG/DTA-SDT Q600 module managed by a Thermal Advantage for Q Series program (v. 5.5. 24), both from TA Instruments. Measurements were made under N_2_ and dry air in a dynamic atmosphere with a flow rate of 50 mL min^–1^, using a sample mass of 7.0 ± 0.2 mg weighed on a thermobalance with an accuracy of ±0.1 μg, temperature range of 25–1000 °C, heating rate of 10 °C min^–1^_,_ and an open α-alumina sample holder.

##### 2.4.4.2 Dynamic Mechanical Analysis (DMA)

The equipment used for the DMA was a DMA Q800 module controlled by the Thermal Advantage Series program (v. 5.5.24), both from TA Instruments. The exact dimensions of the specimens were determined using a Mytutoio micrometer with an accuracy of 0.001 mm and recorded in instrumental control software at the beginning of the measurements. The samples were previously heat treated at 120 °C for 2 h.

##### 2.4.4.3 Glass Transition, *T_g_*

The polyurethane samples in different stoichiometric compositions and the composites containing different proportions of PU and CTS were cut into specimens measuring approximately 33 mm in length, 12 mm in width, and 3 mm in thickness. A single cantilever clamp was used in multifrequency-strain mode. The analysis was performed under the following conditions: heating rate of 3.0 °C min^–1^, temperature range of −60 to 130.0 °C, oscillation frequency of 1 Hz, and amplitude of 20.0 μm.^51,52^

##### 2.4.4.4 Traction Test

Samples were cut into approximate dimensions of 17 mm in length, 12 mm in width, and 2.5 mm in thickness. A single cantilever clamp was used in multifrequency-strain mode under the following conditions: balanced at 25 °C, followed by a 3 min isotherm and force ramp from 1 N min^–1^ to 18 N.

### 2.5 Scanning Electron Microscopy (SEM)

SEM images were obtained using the ZEISS LEO 440 equipment (Cambridge, England) with an OXFORD detector (model 7060), operating with a 15 kV electron beam, 2.82 A current, and 200 pA I probe. The samples were coated with 6 nm gold in a BAL-TEC MED 020 metallizer coating system (BAL-TEC, Liechtenstein) and kept in a desiccator until analysis. The metallization conditions were a chamber pressure of 2.00 × 10^–2^ mbar, a current of 60 mA, and a deposition rate of 0.60 nm s^–1^.

### 2.6 Contact Angle

Contact angle measurements were performed using a C201 Attension Theta Flex optical tensiometer equipped with a Navitar digital camera with 50 optical scans and Attension software. The equipment enables measurements in static mode. Samples were cut into dimensions of 10 mm in length, 10 mm in width, and 2 mm in thickness and were tested in H_2_O solvent.

### 2.7 Swelling Degree (SD)

Samples measuring 10 mm × 10 mm × 2 mm were left in a desiccator for 48 h and then placed in beakers containing 20 mL of H_2_O. At predetermined times, samples were removed, dried lightly with a paper towel, and weighed. Swelling was monitored for up to 72 h. This procedure was adapted from ASTM D570.^53^

### 2.8 In Vitro Cytotoxicity Assays

Cytotoxicity is a parameter for the study of biocompatibility and can be defined as the ability of a carrier system to be administered through a certain route without causing an excessive cytotoxic reaction in the host. According to the International Organization for Standardization (ISO 10993-5), different in vivo and in vitro tests can be applied to determine the cytotoxicity of drug carrier systems.^54^

The L929 fibroblast line was cultivated in a culture flask (75 cm^2^) with Dulbecco’s modified Eagle’s medium (DMEM) supplemented with 10% fetal bovine serum and 1% penicillin/streptomycin at 37 °C in a humified atmosphere and 5% CO_2_. Cells were plated with a density of 1 × 10^6^ cells mL^–1^ and passed three times before the start of the experiments. Cellular assays were performed only when cell growth reached 75–80% confluence on the surface of the flask. All procedures involving cell manipulation were performed in a laminar flow hood under sterile conditions.^54^

When the desired confluence was reached, a cell suspension was carried out at a concentration of 2 × 10^5^ cells mL^–1^. Subsequently, 4 mL of this suspension was pipetted into each well (3.5 cm in diameter) of the six-well plates (Costar) in DMEM with 5% fetal bovine serum. These plates remained in an oven at 37 °C with 5% CO_2_ for 48 h to enable adhesion and confluence of the cell monolayer. Minimum cell viability to perform the assay was 85%. It is essential to observe morphological changes or signs of contamination of the cell culture before starting the assay, which would determine the discarding of the culture. The cultures were examined using an inverted microscope to confirm cellular health, monolayer confluence, and the absence of contamination. Confluence is achieved when the entire area available for growth is occupied and the cells maintain contact with each other.^54^

After 48 h, the culture medium was aspirated and the wells were washed with 2 mL of phosphate-buffered saline (PBS), pH 7.4. Soon after, the PBS was aspirated, and 1 mL of the covering medium was added to each well. This covering medium was composed of 1.8% agar with the addition of 0.01% neutral red dye and 2× concentrated DMEM (1:1 v/v). Both the agar and culture medium remained in a water bath at 40 °C until they were placed in contact with the cell monolayer. The plates remained in the laminar flow hood for 15 min until the solidification of the agar at room temperature. Membranes were cut into discs measuring 0.5 cm in diameter, moistened in the culture medium, and placed with surgical forceps into the center of the well containing agar. For cell control, the first well received only the covering medium. A filter paper disc soaked in DMEM culture medium was used as the negative control, and a filter paper disc soaked in Triton-X was used as the positive control. All membranes were tested in triplicate. The plates were wrapped in aluminum foil to avoid cell damage due to photoactivation of the neutral red dye and placed in an oven at 37 °C with 5% CO_2_ for 24 h. After incubation for 24 h, the wells were observed macroscopically. Cytotoxicity was demonstrated by the formation of a clear halo around the samples and positive controls.^54^ The extent of the bleached area was divided into four quadrants, starting from the disk containing the samples. The quadrants were measured using millimeter transparencies under the plates and calipers, and the areas were recorded.

### 2.9 Cell Adhesion Determined by Immunofluorescence Analysis

The experiment was performed by seeding 30,000 cells/surface in a 100-μL drop to maintain retention to the surface. The experiment was fixed in 4% paraformaldehyde for 10 min, followed by immunofluorescence double staining to test early adhesion (after 4 h) and spreading (after 24 h).^55^

For staining, fixed cells were washed twice with PBS (Gibco, No. 10010023), pH 7.4, and incubated for 10 min with 20 mM NH_4_Cl (Sigma-Aldrich, No. A9434). The samples were then permeabilized for 20 min with Triton X-100, washed again with PBS and labeled for 30 min with phalloidin647 solution (fluorescent at 647 nm) (Thermo Fischer COD.A22287). After washing in PBS, slides were mounted in Fluoroshield with 4′,6′-diamino-2-phenyl-indole (DAPI) (F6057-Sigma-Aldrich). Nucleus staining was read at a wavelength of 405 nm. Images were obtained using excitations at wavelengths of 405 nm (blue), 488 nm (green) and 647 nm (deep red) using a Leica TCS-SPE confocal microscope equipped with lasers (diode 405, diode 488, diode 532 and diode 647) and a galvanometer for displacement in Z from the Multiuser Confocal Microscopy Unit of Institute of Biomedical Sciences of the Federal University of Rio de Janeiro.^55^

## 3 Results and Discussion

### 3.1 Nuclear Magnetic Resonance of Carbon 13 in the Solid-State ^13^C NMR

^13^C NMR spectra were obtained to observe the formation of the urethane bond and changes in chemical shifts of the main PU signals after the addition of CTS during polyol synthesis. The results are displayed in Figures S1 and 4 and the respective peak values are compiled in Table 2.

**Table 2:** Assignment of the Chemical Shifts of the ^13^C NMR Spectra of the PU, CTS, PU90CTS10, and PU50CTS50 Samples

chemical shifts (δ)/ppm															
samples\signal (peak)	C=O (a)	C–N (b″) C=O (a″)	benzene ring (c″, d″, e″, f″, g″)	C=C (j)	C1	C4	glycerol (a′, b′, c′)	(l)	C3, C5	C6	ethylene glycol; triethanolamine;	C2	aliphatic CO (b–h; k, m–q)	CH_3_	(r)
PU	173.3; 170.6	154.6	136.7; 130.2; 119.2	121.3	*	*	76.9; 74.9; 69.1	71.9	*	*	62.8; 59.7; 55.8; 53.2	*	39.9; 34.0; 32.2; 30.0; 23.4;	*	14.6
CTS	174.7	*	*	*	105.2	81.6	*	*	75.5	61.1	*	57.5	*	23.7	*
PU90CTS10	175.7; 173.0; 169.7	154.7	136.6; 130.1; 118.7	*	104.9	83.1	75.0; 65.8; 62.4	71.5	75.0	60.5	62.4; 60.5; 57.3; 52.9	57.3	40.6; 39.4; 29.9; 26.3;	23.4	14.6
PU50CTS50	174.7; 174.3; 170.2	154.5	136.9; 130.2; 119.6; 118.1	122.7	105.0	83.0	75.4	*	75.4	61.0	61.0; 57.7	57.7	40.2; 30.0; 26.0;	23.5	14.7

Qualitatively, the PU spectrum has broad, overlapping groups of peaks in the 25–45, 45–75, and 120–140 ppm regions in addition to well-defined peaks at 15 and 155 ppm. Although different polyols can be used for preparation, ^13^C NMR spectra of polyurethanes are well documented in the literature.^56–58^ The CTS spectra exhibited peaks in the 50–105 ppm region as well as others at 23 and 174 ppm.^32^ For composite polymers containing different percentages of PU and CTS, the presence of both polymers was observed. Depending on the percentage, peaks of PU or CTS were more evident in the spectra, and a mixture of peaks was clearly seen in the PU50CTS50 composite, which was an equimolar mixture.

The assignments in Figure 4 were made according to the respective carbon atoms of the two polymers. The characteristic peaks of urethane bonding (−RNHCOOR′−) can be seen at 170 ppm (C=O) and 155 ppm (C–N). These characteristic peaks of the diisocyanate aromatic portion are observed at 140–120 ppm (C=C). The peaks in the 53.0–62.5 ppm region correspond to glycerol, ethylene glycol, and triethanolamine. The overlapping peaks in the region of 23.0–40.0 ppm are characteristic of carbon atoms in the fatty chain of the aliphatic castor oil. For CTS, carbons in the N-glucosamine and N-acetylglucosamine units were observed in these overlaps and at 175 ppm. The ^13^C NMR studies confirmed the formation of the urethane bond, as well as the presence of CTS in the composite matrix. The spectra are in agreement with similar profiles presented elsewhere.^32,56–58^

**Figure 4:**
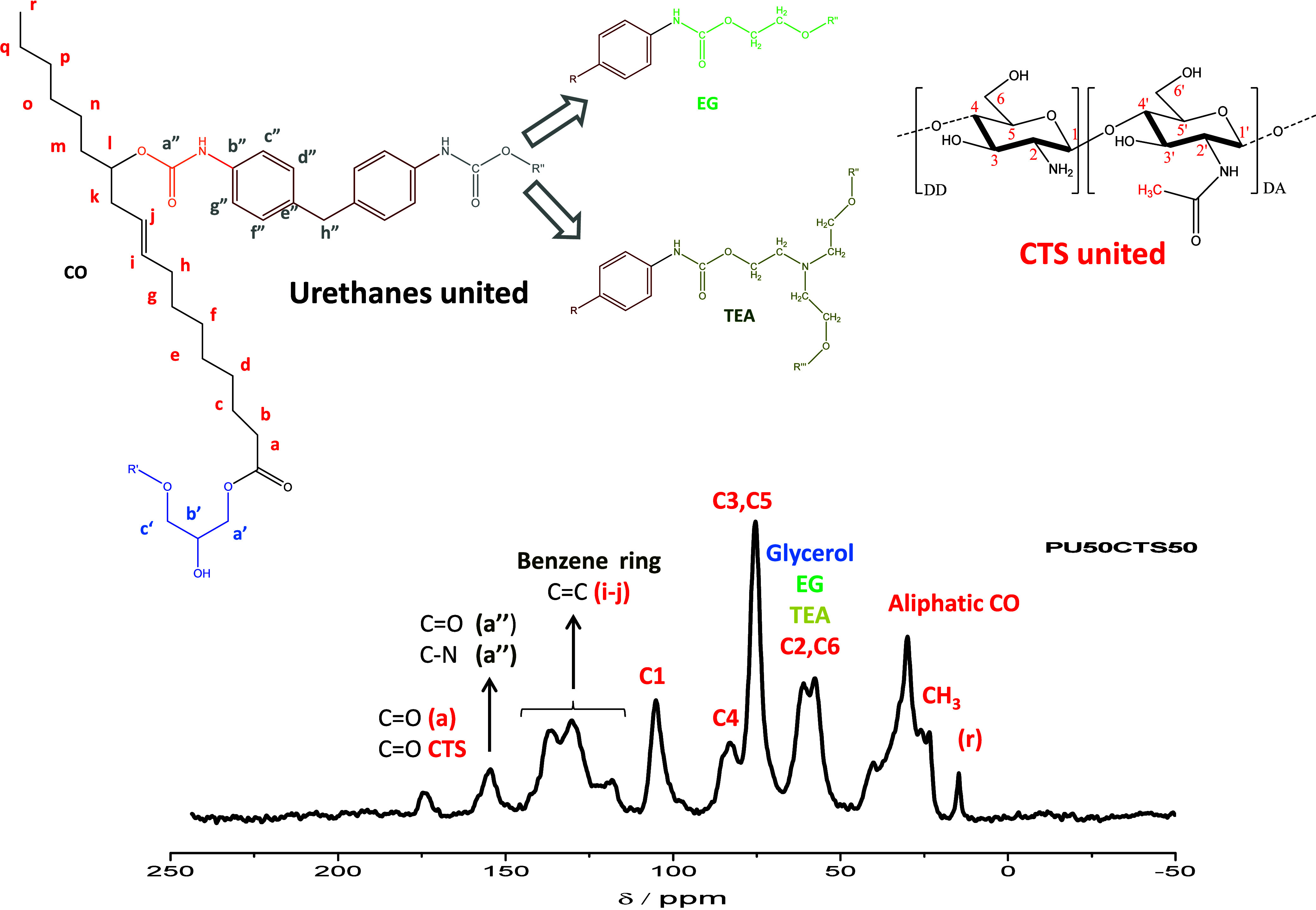
^13^C NMR spectrum of PU50CTS50 and the respective carbon atoms assignments.

### 3.2 Infrared Spectroscopy

FTIR spectra were also obtained to monitor the polymerization and synthesis of the urethane bonds. Figure 5a displays the spectra of the polyol, diphenylmethane diisocyanate (MDI), and stoichiometric polyurethane for the purposes of comparison. Figure 5b shows the PU-CTS composites with different compositions. Band assignments can be seen in Table S1.

**Figure 5:**
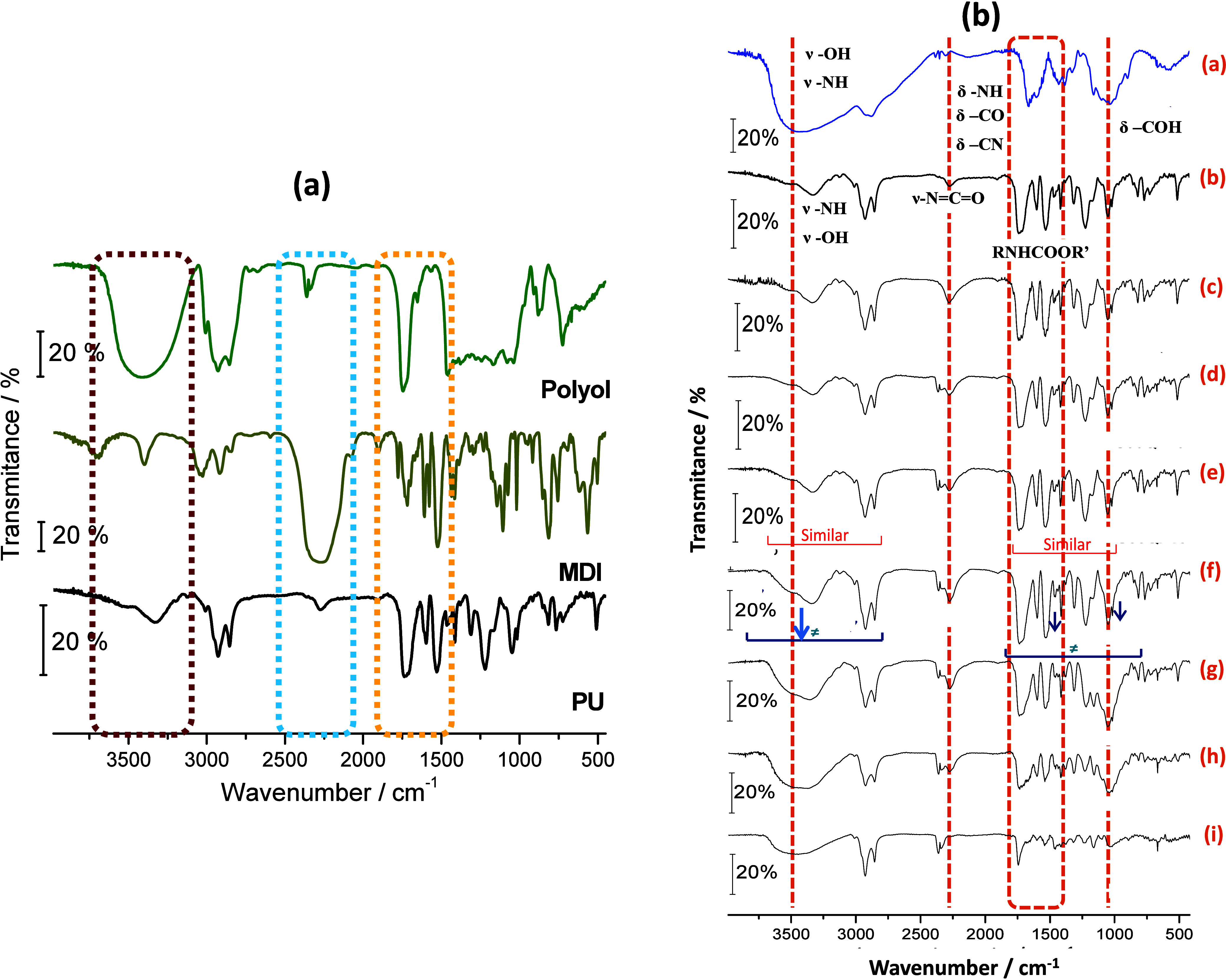
FTIR spectra: (a) comparison of the polyol, diphenylmethane diisocyanate (MDI) and stoichiometric polyurethane PU; (b) comparison of the (a) CTS and (b) PU with the composites formed: (c) PU95CTS5, (d) PU90CTS10, (e) PU85CTS15, (f) PU75CTS25, (g) PU50CTS50, (h) PU25CTS75, and (i) PU5CTS95.

The formation of the urethane polymer was confirmed, as the band referring to the O–H bond at about 3500 cm^–1^ present in the polyol and the band at 2247 cm^–1^ referring to the N=C=O group in the MDI were not observed with the same profile and intensity when compared to those in the PU spectrum. This indicates that the bonds were transformed when the O–H with N=C=O groups chemically interacted, confirming polymerization. Other changes included the absorption bands in the 1750–1500 cm^–1^ region, which were different from the bands seen for the starting reagents due to the formation of the urethane bond, which involves an increase in the absorption intensity and vibration for the C=O and N–H groups.

The CTS spectrum had the characteristic bands of the biopolymer previously reported in the literature (Table S1).^32,59^ Comparing the CTS and PU spectra to those of the PU95CTS5, PU90CTS10, and PU85CTS15 composites, few spectral changes are noticeable, as the percentage of CTS was relatively small. However, when the highest percentage of CTS was used, the PU75CTS25, PU50CTS50, and PU25CTS75 composites had spectra with absorption bands in the 3600 to 3100 cm^–1^ and 1750 to 850 cm^–1^ regions, similar to the CTS spectrum.

### 3.3 X-ray Diffraction

Crystallinity indices were calculated from the diffractograms obtained for the PU, CTS, PU90CTS10, and PU50CTS50 samples shown in Figure 6.^23,50,60^

**Figure 6:**
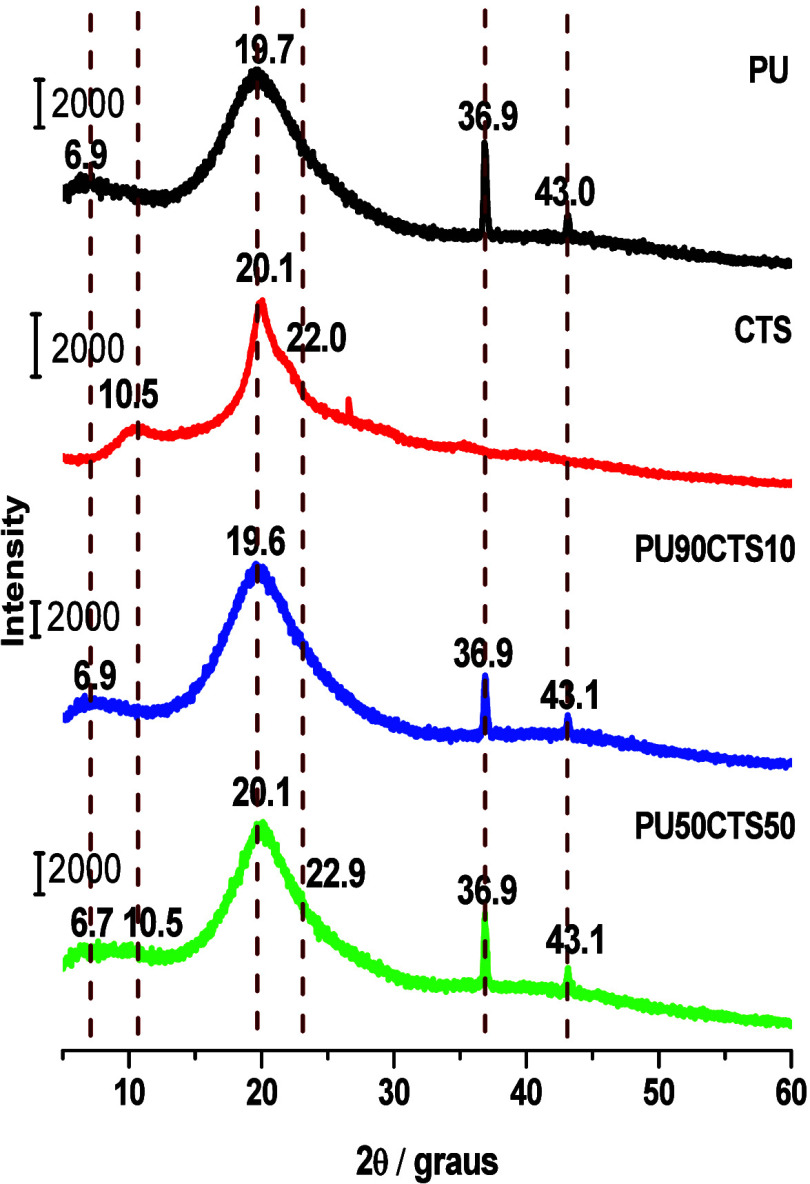
Diffractograms of PU, CTS and composites PU90CTS10 and PU50CTS50.

The crystallinity of the polyurethane samples is related to the peak at 2θ = 19.7°, resulting from ordered regions attributed to the rigid MDI segments and greater packing of the polymer chains.^23,50,60^ In CTS, crystallinity is related to the distribution of the acetylated units of the copolymer and is linked to the peak at 2θ = 20.1°.^32^ These values were calculated based on the intensity of the total area of the diffractogram and the intensity of the crystalline portion, according to eqs 1 and 2. Table S2 displays the results obtained.

$$\%\mathrm{Amorphicity}=[\frac{{\mathrm{Area}}_{\mathrm{total}}-{\mathrm{Area}}_{\mathrm{crystaline}}}{{\mathrm{Area}}_{\mathrm{total}}}]\times 100$$ 1

%Crystalinity=100−%Amorphicity 2

As shown in Table S2, the crystallinity index calculated for PU was 62.2%. CTS added to the system significantly did not affect this index, as only a few changes were found in the results. Although CTS contributed to the crystallinity index, the packing of the more linear regions was affected with the greater amount of CTS distributed in the polyurethane matrix, explaining the small fluctuation in the calculated index values.

The PU, PU90CTS10, and PU50CTS50 samples exhibited four characteristic peaks in the diffractograms, with two broad peaks observed at 2θ equal to 6.9° and 19.7° and two small sharp peaks at 2θ equal to 36.9° and 43.0° not identified. A comparison of the diffractograms reveals that the peak at 2θ equal to 10.5° became more pronounced with the increase in the quantity of CTS in the composition, as seen in the PU50CTS50 sample.

### 3.4 Thermal Characterization

The thermal behavior of PUCTS composites was investigated by TGA/DTG and DTA, with the results presented in Figure 7. The quantitative results obtained for different samples, including mass losses, temperature ranges, and peak temperatures of the events, are summarized in Table S3.

**Figure 7:**
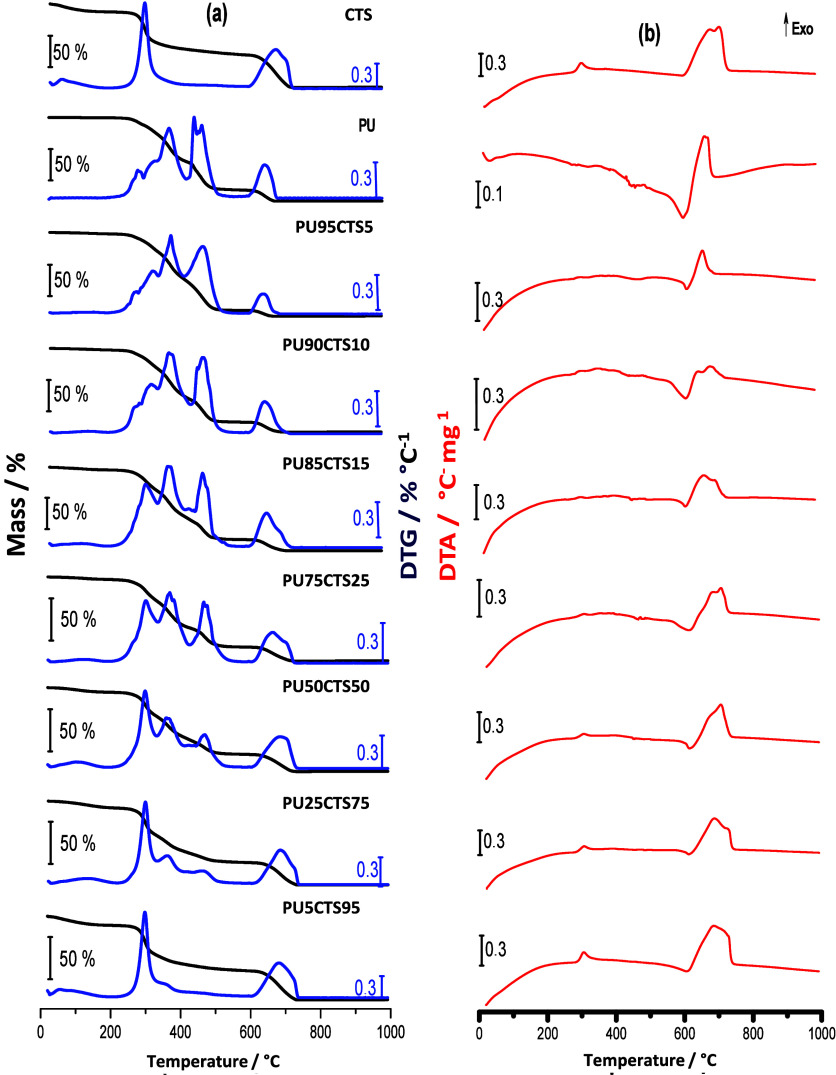
(a) TGA/DTG and (b) DTA curves of composites containing different compositions of PU and CTS. The curves were obtained under an atmosphere of N_2_ and air, sample mass of approximately 7.0 mg, heating rate of 10 °C min^–1^, flow rate of 50 mL min^–1^ and sample support in α-alumina.

As seen in Figure 7a, the TGA/DTG curve for CTS showed three loss-of-mass events (dehydration, decomposition, and the burning carbonaceous materials).^32^ The TGA/DTG curve for PU suggested four mass loss steps: loss of the residual solvent, output of volatile molecules, decomposition of the polymer, and the burning of carbonaceous materials. DTG suggested that these events involve overlapping processes.

Comparing these curves to those of the PUCTS composites, the TGA/DTG curves for the composites containing a higher fraction of PU exhibited a similar profile to the TGA curve for PU, as observed for the PU95CTS5 and PU90CTS10 composites. However, when the fraction of CTS in the composite increased, the TGA/DTG curve began to show characteristics closer to those of CTS, as occurred for the PU85CTS15 and PU75CTS25 samples (Figure 7a). In these cases, an increase in the DTG peak was found in the region of 300 °C. The third (369 °C) and fourth (451 °C) events involved lower mass losses, as demonstrated by the intensities of their respective DTG peaks. The comparison of the second, third, and fourth loss-of-mass events revealed DTG curves with similar peaks. The TGA/DTG curves for PU50CTS50 and PU25CTS75 show the clear presence of CTS and a decrease in mass losses in the third and fourth stages, whereas the PU5CTS95 curve was similar to the CTS curve.

As shown in Table S3, the PUCTS composites exhibited an increase in thermal stability, with the decomposition process starting at approximately 200 °C, while CTS and PU were thermally stable up to 193 and 171 °C, respectively, after the loss of the solvents. This suggests greater stability in the composites, likely due to chemical interactions between the functional groups of the two polymers.

All PUCTS composites had similar DTA profiles up to 200 °C. From 200 °C onward, the PU95CTS5 and PU90CTS10 composites exhibited events similar to those seen in the DTA curve for PU up to 700 °C. However, with the increase in the quantity of CTS in the composite, the DTA curves for PU85CTS15, PU75CTS25, PU50CTS50, PU25CTS75 and PU5CTS95 exhibited similar events to those of the DTA curve for CTS. Peak temperatures are shown in Table S3.

The glass transition (*T_g_*) is an important feature to guide applications of polymeric materials that can be assessed by dynamic mechanical analysis (DMA). The PU-CTS composites containing different percentages of the components were submitted to DMA and the resulting curves are given in Figure 8. These curves represent changes in the storage (*E*′) and loss (*E*″) moduli as a function of temperature, in addition to the tan-δ curves, which is the relationship between these moduli, the maximum of which corresponds to the glass transition temperature.

**Figure 8:**
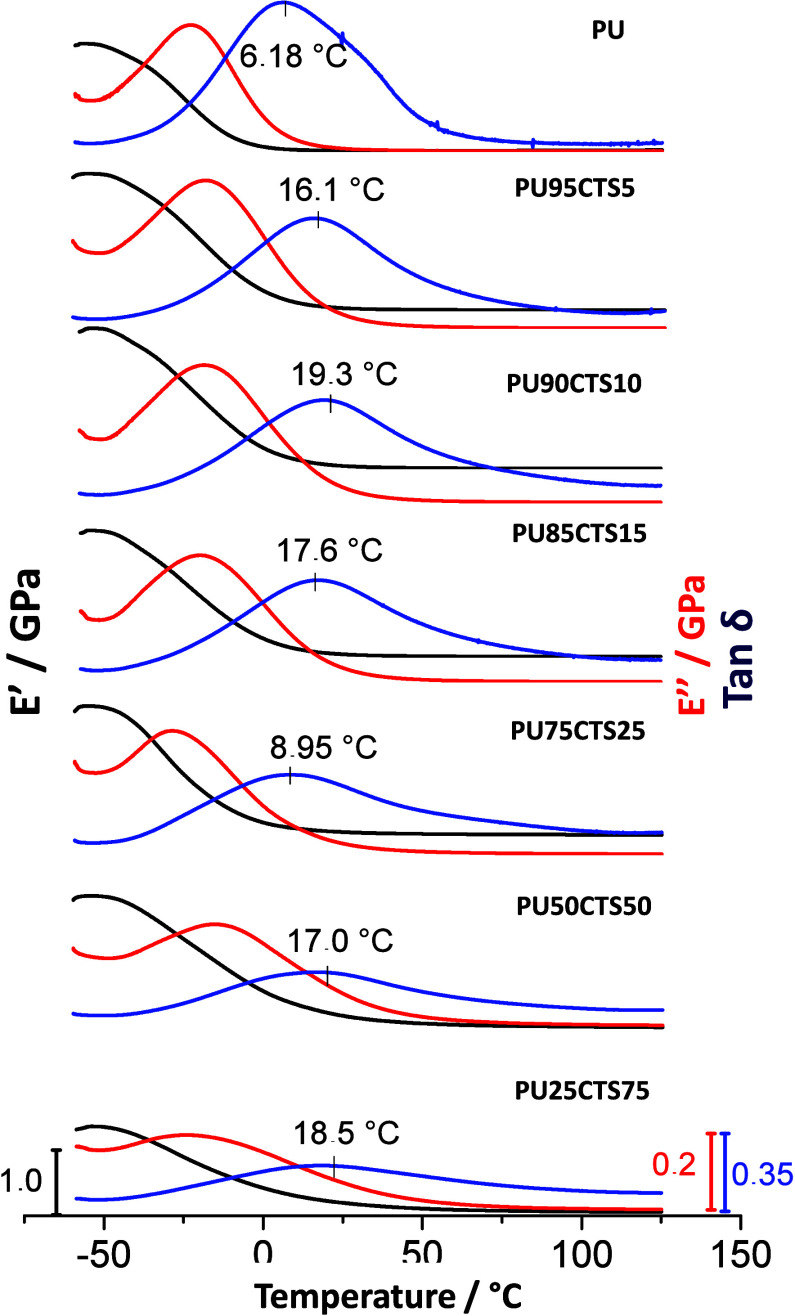
DMA curves for the storage modules, *E*′; of loss *E*″, and tan δ for the PUCTS composites in different percentages.

As seen in Figure 8, the increase in the quantity of CTS in the composite resulted in a shift in the tan delta peak to higher values. Thus, PU in the stoichiometric proportion had a *T_g_* of 6.18 °C, while the composites had glass transition temperatures ranging from 8.0 to 19 °C. This increase was attributed to interactions between functional groups of the polymers and is in agreement with what was seen in the TGA curves, with an increase in thermal stability in the composites, resulting from greater rotation difficulty of the segments of the polymer chain in the presence of CTS, which also leads to an increase in the stiffness of the material.

On the other hand, the intensity of the tan delta peak decreased with the increase in the percentage of CTS. The PU25CTS75 sample had the lowest intensity, as seen by the tan delta peak, presenting a softer characteristic, whereas the highest intensity was found for PU95CTS5, which exhibited greater stiffness.

Table 3 presents the peak values of the storage and loss moduli and peak temperatures of the tan delta curves. The changes in the glass transition temperature directly imply the processing of the material. Thus, it appears that the composites had higher glass transition temperatures than polyurethane, in addition to contributing synergistic effects due to the properties of CTS.

**Table 3:** Values Obtained from DMA Curves for Different Percentages of PUCTS Polymer Composites

samples	module		peak
	*E*′ (GPa)	*E*″ (GPa)	*T*_*g*_ (°C)
PU	1.89	1.24	6.18
PU95CTS05	1.76	0.13	16.1
PU90CTS10	1.72	0.13	19.3
PU85CTS15	1.41	0.14	17.6
PU75CTS25	1.42	0.14	8.95
PU50CTS50	1.45	0.10	17.0
PU25CTS75	1.23	0.08	18.5

### 3.5 Traction Test

The stress and strain curves obtained at 25 °C suggest different mechanical behaviors depending on the CTS content in the composites, as shown in Figure 9.

**Figure 9:**
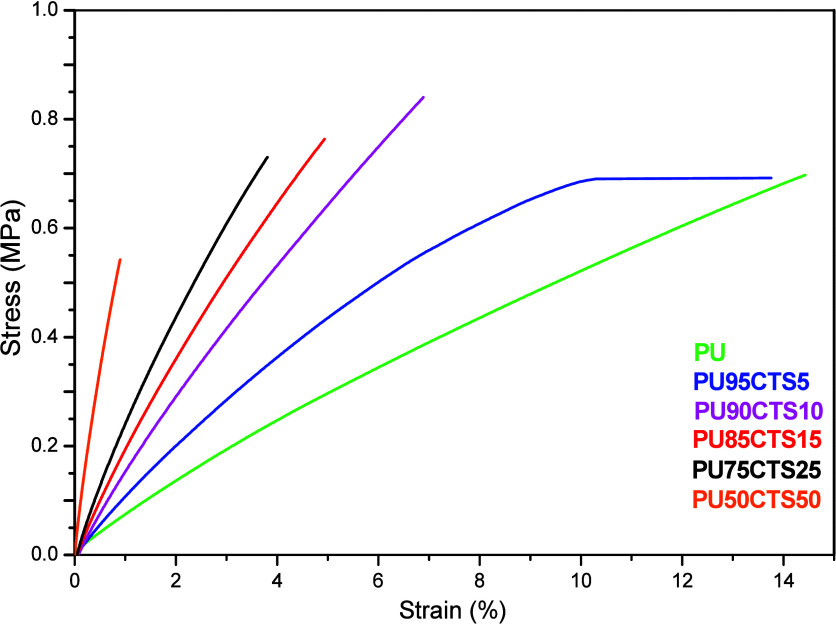
Stress/strain curve obtained at 25 °C for the PU and PUCTS composites in different percentages.

The curves in Figure 9 were obtained above the glass transition temperatures of all samples. The PU sample suffered deformation of around 14% supporting a stress variation of 0.69 MPa. The presence of 5% CTS (m/m) in the material did not significantly alter the mechanical property, but the increase to 10% of the biopolymer promoted an increase in tension to 0.83 MPa (PU90CTS10), which then began to decrease with the increase in the CTS content, reaching 0.54 MPa in the PU50CTS50 sample.

The slopes of the stress–strain curves changed until reaching the yield limits (σ). Table 4 displays these values, along with the strain values at the yield limit (% ε), elastic moduli, or Young’s modulus (*E*), in addition to the values of the varied size of each sample (displacement).^6,16^ The viscoelastic behavior of PUCTS is demonstrated by the curves shown in Figure 9. These results suggest that the composites have thermoset characteristics, with small strains and elongation as well as low % *E*.

**Table 4:** Values of Tension/Strain Tests, Carried Out at 25 °C for the PUCTS Composites in Different Compositions

sample	σ max/MPa	%/(ε)	*E*/MPa	displacement Δ*l*/mm
PU	0.69	14.4	0.04	1.60
PU95CTS5	0.68	10.2	0.06	2.00
PU90CTS10	0.83	6.89	0.12	1.10
PU85CTS15	0.76	4.94	0.15	0.83
PU75CTS25	0.73	3.81	0.19	0.61
PU50CTS50	0.54	0.90	0.60	0.16

### 3.6 Scanning Electron Microscopy

The morphology of the PU, CTS, PU75CTS25, PU50CTS50, and PU25CTS75 samples was investigated using SEM. The micrographs at a magnification of 100× are shown in Figure 10. The CTS sample had smooth plate structures with dimensions above 100 μm, whereas the PU had a uniform surface with roughness and circular points with diameters smaller than 200 μm uniformly distributed in the sample, as indicated by the green arrow, highlighting empty spaces attributed to the outlet of gases formed during the polymerization reaction. The PU75CTS25, PU50CTS50, and PU25CTS75 composites had plate structures randomly distributed on the surface, which correspond to the presence of CTS (yellow arrows), in addition to cavities (green arrows), resulting from the release of gases during the reaction. For PU25CTS75, in which the quantity of CTS was greater than that of polyurethane, PU was found between the CTS plates. The irregular surface, with large gaps and cavities, may be related to the absence of CTS, which would not be fixed in the matrix.

**Figure 10:**
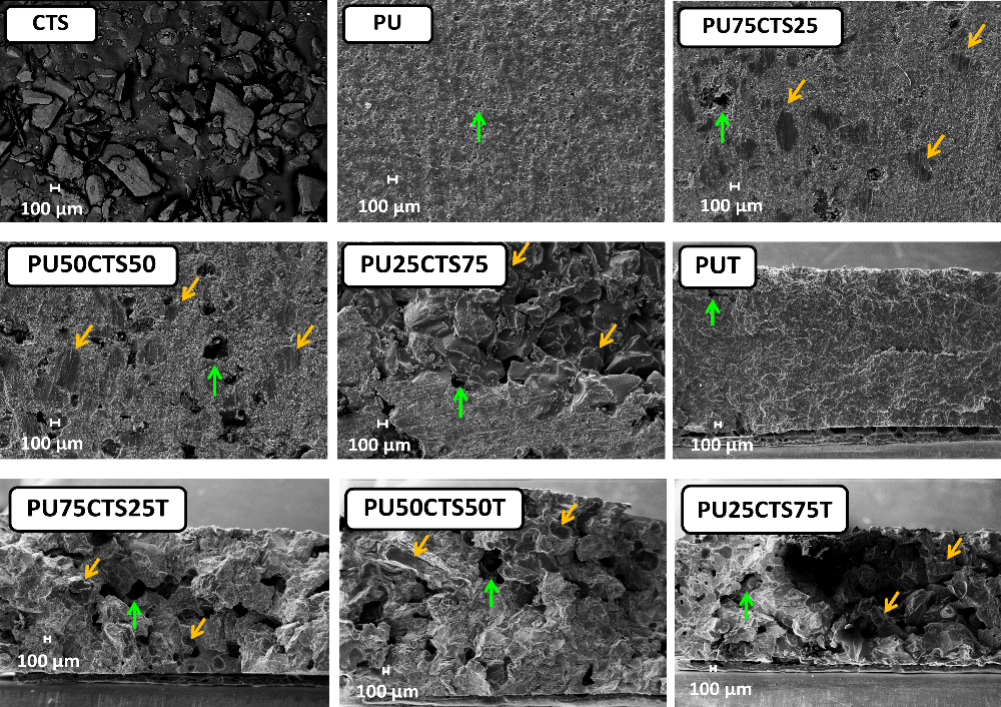
Scanning electron micrographs of the fractured surfaces of the solid samples of PU, PU75CTS25, PU50CTS50 and PU25CTS75, CTS powder, and fractured samples with named with “T” in the end, at a magnification of 100×.

To observe the interior of the specimens, images were also obtained of the fractured faces of the PU, PU75CTS25, PU50CTS50 and PU25CTS75 samples. In the case of PU, the polymer had a compact, uniform lamellar-type structure in the interior portion and a crack on the edge. For PU75CTS25, the presence of CTS was confirmed by randomly arranged particles in the PU polymeric matrix. The cavities with diameters >100 μm indicated by the green arrows are likely due to the formation and release of gases during the polymerization reaction. The structure is compact and uniform. The increase in the quantity of CTS in the PU polymeric matrix, as in PU50CTS50 and PU25CTS75, led to an increase in the diameter of the cavities while maintaining the internal roughness and random distribution of CTS in addition to promoting a less uniform appearance of the material.

### 3.7 Contact Angle Measurements

The contact angle (θ) is defined as the angle formed when a liquid is deposited on the surface of a solid or another liquid and may be θ < 90°, θ = 90° or θ > 90°.^61–63^

A θ less than 90° corresponds to a hydrophilic solid, on which the liquid tends to spread, wetting the surface. When above 90°, the liquid does not spread, remaining in the shape of a drop. The shape of the drop is related to surface tension and external forces, such as gravity.^61–63^

Figure 11 displays the results of contact angle measurements for PU and the PUCTS composites using water. The experiments were conducted in the static regime, and the angles were calculated based on the Young–Laplace Equation. Both the PU and composites with different percentages of PUCTS had average left and right angles above 90°, demonstrating the hydrophobic nature of the materials. The contact angles are displayed in Table S4.

**Figure 11:**
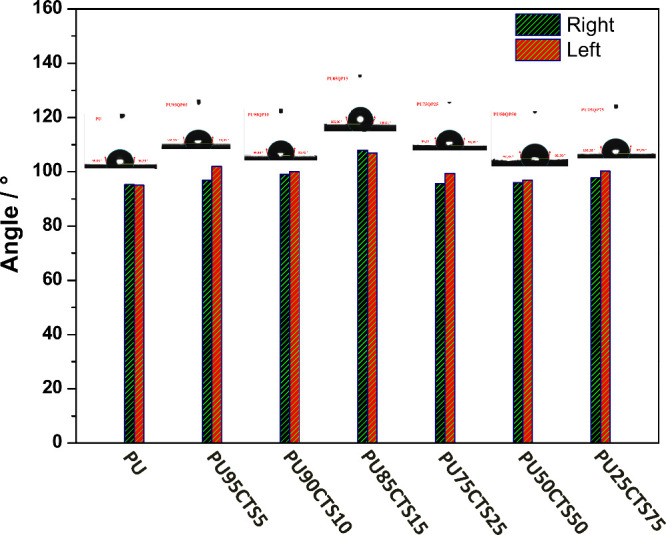
Contact angle measurements of samples PU, PU95CTS5, PU90CTS10, PU85CTS15, PU75CTS25, PU50CTS50, and PU25CTS75.

The presence of chitosan in the polyurethane matrix did not considerably increase the contact angle of the PUCTS composite materials. A small increase in hydrophobicity was found, regarding PU itself, but the irregularity on the polyurethane surface was increased, with microscopic interstices/cavities revealed by SEM images, which were filled with air. As the equipment performs the measurement considering the Young–Laplace equation, that is, the measurement for a homogeneous smooth surface, such irregularity can promote the same influence on the results. Actually, it is possible to consider that these results are statistically similar if one takes into account the error bars related to the individual measurements (Table S4 in Supporting Information). The unexpected results for PU85CTS15 can be explained by the same heterogeneity in the sample.

Comparing the values obtained from the contact angle measurements of PU and the PUCTS composites to results found in the literature on polyurethanes prepared from castor oil, similarities can be seen, with hydrophobic characteristics and angles above 90°.^64^ For biological applications, understanding the surface properties of the synthesized composite, such as the contact angle and wettability, is of paramount importance, as it enables the determination of how biological fluids may interact and behave when they are in contact with the material.

### 3.8 Swelling Degree

The PU and PUCTS composites were subjected to swelling measurements to investigate their ability to absorb water, which is an important parameter in biological applications. These measurements were made following the ASTM D570 standard.^53^ Figure 12 displays the average values obtained.

**Figure 12:**
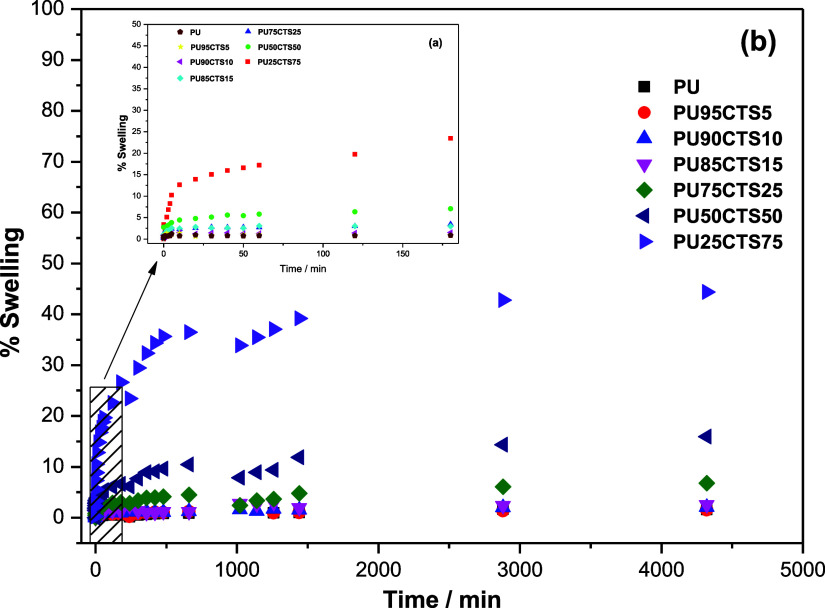
Swelling curves of PUCTS samples at different percentages monitored for 72 h and inset showing the period of 3 h.

The percentage of H_2_O mass absorbed was calculated from eq 3.

$$\%\mathrm{Swelling}=\frac{m_{\mathrm{final}}-m_{\mathrm{initial}}}{m_{\mathrm{initial}}}\times 100$$ 3

where *m*_final_ is the final mass and *m*_initial_ is the initial mass of the sample.

The insert in Figure 12 shows that the samples with the highest CTS content (PU25CTS75, PU50CTS50 and PU75CTS25) absorbed a higher percentage of H_2_O after three hours of monitoring, while the samples with a lower CTS percentage (PU85CTS15, PU90CTS10, PU95CTS5) and PU absorbed approximately 2.5% of H_2_O. This is reasonable, as chitosan has considerable water absorption capacity. After 72 h, higher quantities of CTS led to greater H_2_O absorption. The PU25CTS75, PU50CTS50 and PU75CTS25 samples absorbed approximately 45%, 16% and 7%, respectively, while absorption in the other samples remained around 2.5%.

Theoretically, stoichiometric PU has all–OH groups reacted with diisocyanate, giving the material a hydrophobic nature. However, when the CTS was mixed with the polyol, the hydrophilic nature of the material increased, as hydrophilic groups are found in the biopolymeric chain, enabling greater diffusion of H_2_O, interaction, and swelling. This behavior was observed with the increase in the quantity of CTS.^6,16^

Although the contact angle measurements did not reveal significant differences between the hydrofilicity of the composites, one must consider that this is an instant measurement and sweeling studies are more reliable in order to evaluate the interactions of the sample and the water.

### 3.9 Cytotoxicity Assay

The method employed in the cytotoxicity assays enabled classification of the samples into groups according to the size of the halos formed from the cells. The qualitative classification was made based on the quantitative parameter (halo size), as shown in Table 5.^65^

**Table 5:** Cytotoxicity Degrees for Classifying Samples

degree	cytotoxicity	cytotoxicity zone
0	absence	absence of bleaching under the sample
1	light	bleaching zone limited to the area under the sample
2	mild	bleaching zone from the sample up to 0.5 cm
3	moderate	bleaching zone from the sample between 0.5 to 1.0 cm
4	severe	bleaching zone greater than 1.0 cm

It is well established in the literature that experiments with cell lines facilitate the reproducibility of results. Thus, cell lines are commonly used in cytotoxicity assays.^66^ The cellular assays were performed with L929 rat fibroblasts. This lineage is recommended by ISO 10993-5 and is commonly used in biocompatibility studies.^67^

Considering current discussions in the scientific community regarding the use of animals for testing substances, in vitro testing commonly precedes in vivo testing.^66^ Methods for investigating cytotoxicity are reproducible and relatively inexpensive. Despite this, in vitro results are not exactly the same as in vivo results. Nonetheless, a material with high cellular cytotoxicity will likely be toxic to tissues, requiring in-depth studies in more advanced stages of assessing the material.^66^

Before the experiment was started, cell viability was assessed by verifying the presence of more than 90% viable cells. Next, we qualitatively analyzed the cytotoxic levels for liquid crystalline systems. The bleached extent of areas per quadrant (*Q*) is presented in Table S5. The quantitative mean cytotoxicity values obtained for each system were interpreted according to Table 5 (classification of the degree of cytotoxicity) to obtain the qualitative results displayed in Table 6. The composites with the lowest cytotoxicity when tested on fibroblast cells were PU90CTS10, PU85CTS15, PU50CTS50, and PU25CTS75, which exhibited slight cytotoxicity, based on Table S5, whereas the other samples exhibited mild cytotoxicity. Thus, these materials have properties that suggest promising future applications in the preparation of medical devices.

**Table 6:** Qualitative Cytotoxicity Results Obtained for the Systems Studied

system	final halo average (cm)	cytotoxicity
control +	1.2	severe
control –	0	absence
PU	0.3	light
PU95CTS5	0.4	mild
PU90CTS10	0.2	light
PU85CTS15	0.3	light
PU75CTS25	0.4	mild
PU50CTS50	0.3	light
PU25CTS75	0.3	light

### 3.10 Cell Adhesion

The interaction of the material with cells is necessary for the regenerative processes. In this study, cell adhesion was investigated by using fluorescence microscopy after contact with different surfaces. Good cell adhesion usually occurs in two stages: early adhesion (at around four h of contact) and spreading (at 24 h).

To perform fluorescence analysis, it was necessary to select fluorescent markers. These are ligands for specific molecules of interest conjugated to a fluorochrome, which is a photosensitive compound used to detect these molecules. In the initial analyses, however, we found that the PU, PU90CTS10, and PU50CTS50 samples exhibited autofluorescence (strong green at 488 nm, strong orange at 546 nm, and weaker blue at 350 nm), which limited the choice of fluorochromes that emit at these wavelengths, making some markers unusable.

A marker for globular proteins was used to overcome this problem, specifically, phalloidin, which binds to cytoskeletal actin present in eukaryotic cells. The fluorochrome conjugated to phalloidin fluoresces at 647 nm (near-infrared region) and does not interfere with the autofluorescence of the sample. Consequently, the experiments were conducted using two markers: (i) phalloidin conjugated with a red-emitting fluorochrome and (ii) 4′,6-diamidino-2-phenylindole (DAPI), which emits blue fluorescence. A third green fluorescence was also present due to the autofluorescence of the sample.

Figure 13 shows the confocal microscopy images from the immunofluorescence analysis of the PU, PU90CTS10, and PU50CTS50 samples taken after four and 24 h. Figure 13a shows merged images with 366 (blue) and 546 nm (red) filters in the first column, while the second column includes the addition of a 488 nm (green) filter. Therefore, the following can be seen in Figure 13a,b: the sample surface is shown in green; cell nuclei appear in blue, and the cytoskeleton of L929 cells outlining the cell boundaries is shown in red, indicating the area occupied by the cells on the material. A greater surface area that a cell occupies on the sample (known as cell spreading) indicates that the material is more suitable for biomedical applications.

**Figure 13:**
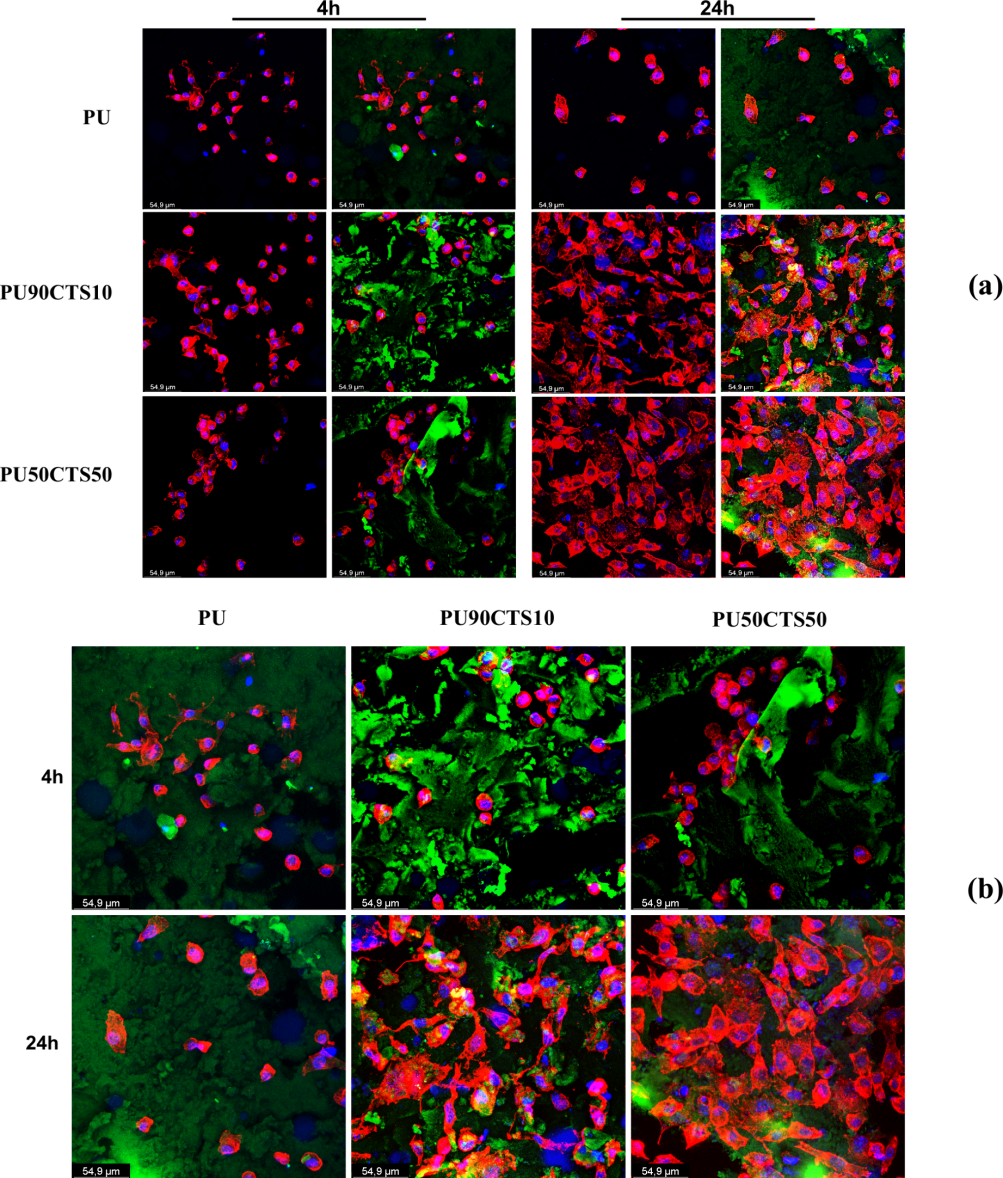
Images obtained from a confocal microscope of the immunofluescence results of the PU, PU90CTS10, and PU50CTS50 samples, when cell adhesion was tested after L929 cultivation, using the cytoskeletal actin marker. (a) The first column includes 366 nm (blue) and 546 nm (red) filters, while the second column features the addition of a 488 nm (green) filter; (b) merge of all the filters.

Figure 13a,b show autofluorescence of all surfaces, which was more intense on the PU90CTS10 and PU50CTS50 samples, while a lower fluorescence was found in the PU sample.

Moreover, early adhesion seems to have been similar in all samples, whereas differences were found after 24 h of adhesion. The surfaces of the PU90CTS10 and PU50CTS50 samples exhibited an increase in the number of cells and greater cell spreading compared to cells adhered to the PU surface, which were fewer and had a more rounded shape. This behavior suggests that cells not only adhere to the modified surfaces but also proliferate, indicating the potential of these compositions as a basis for developing biomaterials with cell regeneration properties, which is the focus of this study.

The greater cell adhesion in the PU90CTS10 and PU50CTS50 samples may be linked to the chemical groups present in membrane proteins, particularly the lipid bilayer (side groups of amino acids with polar characteristics), which have a charge density that facilitates secondary interactions (van der Waals forces and hydrogen bonding) with groups present in the CTS chain structure (−NH_2_ and −OH groups). In contrast, the PU sample has a nonpolar surface, preventing interactions with the polar groups found in the plasma membrane of the cell. These results also indicate that the PU sample (without the presence of CTS) did not induce cell proliferation, making it potentially interesting for use as a raw material for biomaterials with healing properties.

## 4 Conclusion

The polyurethane composites formed from vegetable oil-based polyol MDI and CTS were successfully prepared. Based on the stoichiometric PU, different composites between PUCTS were studied.

These materials were characterized using spectroscopic, thermal, morphological, and mechanical methods, exhibiting thermal and mechanical properties that varied depending on the PU:CTS composition.

Morphological studies showed that the PU surfaces were homogeneous and compact with small holes resulting from the release gases formed during polymerization. The presence of CTS gradually changed the morphology with the appearance of smooth plates as the quantity of the biopolymer increased.

Physical tests involving contact angle measurements and the degree of swelling were carried out to determine the hydrophilicity and H_2_O absorption potential of the materials. PU and its composites exhibited hydrophobicity, whereas an increase in the quantity of CTS enhanced the absorption potential.

According to the biological tests, the materials with the lowest cytotoxicity to fibroblast cells were PU90CTS10, PU85CTS15, PU50CTS50, and PU25CTS75, which exhibited slight cytotoxicity, while the other materials exhibited mild cytotoxicity.

In the cell adhesion tests, the PU90CTS10 and PU50CTS50 samples promoted the adhesion of fibroblast cells at four and 24 h, whereas no adhesion was found for stoichiometric PU.

The polyurethane exhibited flexibility with a glass transition at around 16 °C, which is a useful property for future molding when preparing a specific shape. Furthermore, the properties demonstrated by these materials are attractive for future applications in the field of biomaterials as a polymer prepared from renewable sources such as castor oil.

## Supplementary Material



## Notes

Figure S1 shows the ^13^C NMR spectra comparison between PU, CTS and PU90CTS10 and PU50CTS50, helping to identify the mixture of polymers and formation of urethane bond. The main information was discussed and referenced in the manuscript in the corresponding sections; however, the values obtained in measurements can be seen in Table S1 – FTIR, Table S2 – XRD, Table S3 – TGA/DTG/DTA, Table S4 – contact angle, and Table S5 – cytotoxicity assay. Such tables present one description of content. The data corroborated the observations extracted from FTIR and XRD spectra as well as TGA/DTG/DTA curves and biological assay of PUCTS composite materials prepared from derived castor oil polyol, MDI, and chitosan (PDF)
