# Supplementary material for: Synthesis, Characterization, and Biological Studies of Biopolyurethane-Chitosan Composites Based on Diphenylmethane Diisocyanate and Polyol Derived from Castor Oil for the Development of Biomaterials for Topical Use
Source: ACS Omega. 2025 May 22;10(21):22036–51. doi: 10.1021/acsomega.5c02165 (PMC12138712; doi:10.1021/acsomega.5c02165)
Supplement: Supplementary file 1 [file ao5c02165_si_001.pdf]

Supporting information

Synthesis, characterization and biological studies of bio-polyurethane-chitosan composites based on diphenylmethane diisocyanate and polyol derived from castor oil for the development of biomaterials for topical use

Ricardo dos Santos Medeiros[1], Ana Paula Garcia Ferreira[1], Carolina. K. Sanz[2], Sara Gemini Piperni[2], Kaio Pini Santos[3], Marlus. Chorilli[3], Wagner Luiz Polito[1], Tiago Venâncio[4], Éder Tadeu Gomes Cavalheiro[1]*

(1) Instituto de Química de São Carlos, Universidade de São Paulo,

Av. Trabalhador São-carlense, 400, 13566-590, São Carlos, SP, Brasil.

(2) Laboratório de biotecnologia, bioengenharia e biomtariais nanoestruturados (LabeN), Instituto de Ciências Biomédicas, Universidade Federal do Rio de Janeiro,

Campus Cidade Universitária, Rio de Janeiro, RJ, 21941,

(3) São Paulo State University - School of Pharmacuetical Sciences

- Rodovia Araraquara Jaú, Km 01 - s/n –

Campos Ville- 14800-903 - Araraquara/SP

(4) Departamento de Química- DQ, Universidade Federal de São Carlos

Rodovia Washington Luis s/n Km 235,13565-905, São Carlos, SP, Brasil

Campus São Carlos

**Corresponding author:*

Email:cavalheiro@iqsc.usp.br

Tel: +55 +16 33738197

Supplementary Figures

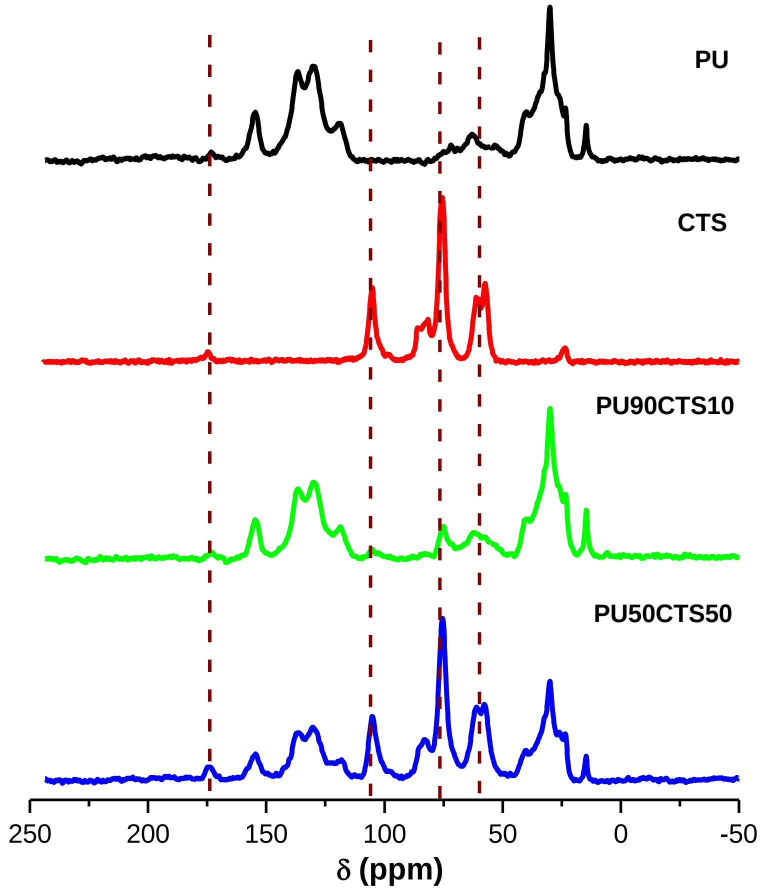

Figure S1. Comparison of ^{13}C NMR spectra of PU, CTS, PU90CTS10 and PU50CTS50.

Supplementary Tables

Table S1. Band assignment of the FTIR spectra of the Polyol, MDI, PU and CTS samples

Wavenumber / cm^{-1}	Assigment*
**Polyol**	
3415	ν -OH
3009, 2929 e 2852	ν –CH_2
1747	δ –C=O
1464	δ – CH_2, CH_3 and C-N
1080	δ – C-O-C
**MDI**	
3126 and 3051	ν –C-H.
2912 and 2841	ν –CH_2
2247	R-N=C=O
1716	ν –C=O
1610	δ –C=O
1577	δ-C=N
1537	δ-N-H
**CTS**	
3600-3100	ν O-H and ν N-H
2916 and 2877	ν C-H
1658	δ C=O
1600	δ N-H
1549	δ C-N
1425	δ C-N
1381	CH_3
1319	δ C-N
1153	ν C-O-C
1101 and 1029	δ C-OH
889	δ -C-H
**PU**	
3550 to 3100	ν -OH
2937 and 2854	ν –CH_2
1751	δ –C=O
1462	δ – CH_2, CH_3
1095	δ – C-O-C

* ν = stretch; δ = deformation.

Table S2. Amorphicity and crystallinity index data obtained from diffractograms for samples PU, CTS, PU90CTS10 and PU50CTS50 and peaks

Samples	Peaks / 2θ/°	Amorphicity	*I_{Cr} (%)
**CTS**	10.5;20.1;22.0	53.2	46.8
**PU**	6.9;19.7;36.9;43.0	37.8	62.2
**PU90CTS10**	6.9;19.6;36.9;43.1	36.4	63.6
**PU50CTS50**	6.7;10.5;20.1;22.9;36.9;43.1	39.2	60.8

*I_{Cr} = Crystallinity Index

Table S3. Data obtained from the TGA/DTG and DTA curves of the CTS and PU samples and the PUCTS composites

Sample	Events (N_2 e ar atmospheres)	TGA/DTG		DTA
		Temperature / °C	Mass loss/ %	Peak temperature/°C
CTS	1ª loss	21.04-193.7	9.53	59.59 (↓);
	2ª loss	193.7-588.4	51.0	282.3 (*↓); 305.5 (*↑)
	3ª loss	588.4-739.7	38.7	602.1 (↓); 683.1 (↑); 711.1 (↑)
	Residue	950.1	0.77	-
PU	1ª loss	17.56-171.5	0.22	38.15 (↓); 72.69 (↓)
	2ª loss	171.5-294.5	8.70	277.5 (↓); 291.8 (↓)
	3ª loss	294.5-420.9	43.6	326.9 (↓)
	4ª loss	420.9-558.1	33.4	434.7 (↓); 454.4 (↓); 463.9 (↓)
	5ª loss	558.1-692.6	13.5	604.5 (↓);669.4 (↑); 677.7 (↑)
	Residue	950.1	0.53	-
PU95CTS5	1ª loss	19.74-188.3	1.64	56.16 (↓);
	2ª loss	188.3-280.4	5.78	265.0 (↓);
	3ª loss	280.4-413.6	48.1	283.5 (↓); 322.8 (↓); 377.0 (↓);
	4ª loss	413.6-588.4	37.3	471.7 (↓);
	5ª loss	588.4-706.0	6.95	615.2 (↓); 661.6 (↑); 689.6 (↑);
	Residue	950.1	0.42	-
PU90CTS10	1ª loss	21.75-197.0	1.92	48.37 (↓)
	2ª loss	197.0-289.2	7.45	269.2 (↓); 287.0 (↓)
	3ª loss	289.2-433.7	51.6	322.2 (↓); 368.6 (↓); 391.3 (↓);
	4ª loss	433.7-576.3	26.4	444.9 (↓); 465.7 (↓);
	5ª loss	576.3-735.6	11.9	612.8 (↓); 648.5 (↑); 686.1 (↑);
	Residue	950.1	0.54	
PU85CTS15	1ª loss	19.58-195.7	3.25	41.39 (↓)
	2ª loss	195.7-285.1	7.49	269.8 (↓);
	3ª loss	285.1-416.9	48.6	285.9 (↓); 323.4 (↓); 360.9 (↓); 385.3 (↓);
	4ª loss	416.9-584.3	27.0	454.4 (↓); 505.6 (↓);
	5ª loss	584.3-748.4	13.4	610.4 (↓); 665.8 (↑); 697.4 (↑);
	Residue	950.1	0.62	-

-continue-

-continuation-

**PU75CTS25**	1ª loss	20.89-198.4	3.12	56.18 (↓)
	2ª loss	198.4-283.8	7.33	261.4 (↓); 283.5 (↓)
	3ª loss	283.8-426.3	50.0	323.4 (↓); 366.3 (↓); 403.2 (↓).
	4ª loss	426.3-585.7	22.7	463.9 (↓); 476.4 (↓)
	5ª loss	585.7-736.3	16.4	613.4 (↓); 680.1 (↑); 705.7 (↑);
	Resíduo	950.1	0.49	-
**PU50CTS50**	1ª loss	20.49-196.4	5.53	52.45 (↓);
	2ª loss	196.4-272.3	4.27	-
	3ª loss	272.3-410.9	49.4	282. 9 (↓); 305.6 (↑); 324.6 (↓);
	4ª loss	410.9-583.7	20.0	449.6 (↓); 453.8 (↓); 482.2 (↓)
	5ª loss	583.7-739.7	20.3	614.0 (↓); 670.1 (↑); 705.1 (↑);
	Resíduo	950.1	0.54	-
**PU25CTS75**	1ª loss	21.89-220.6	8.40	61.38 (↓)
	2ª loss	220.6-269.7	2.98	-
	3ª loss	269.7-433.7	51.5	279.9 (↓); 306.1 (↑); 401.4 (↓);
	4ª loss	433.7-590.4	10.0	455.6 (↓); 484.2
	5ª loss	590.4-743.0	26.5	612.2 (↓); 687.9 (↑); 724.2 (↑);
	Resíduo	950.1	0.59	-
**PU5CTS95**	1ª loss	19.81-206.5	10.8	62.57 (↓)
	2ª loss	206.5-438.4	48.0	281.7 (↓); 306.1 (↑);
	3ª loss	438.4-589.7	5.77	441.3 (↓)
	4ª loss	589.7-743.0	34.6	605.1 (↓); 685.5 (↑); 715.2 (↑); 727.2 (↑);
	Resíduo	950.1	0.70	-

* ↓ = (endothermic) ↑ = (exothermic)

Table S4. Contact angle values obtained for PUCTS samples

**Sample**	**Right angle**	**Left angle**
PU	95 ± 0.$_{4}$°	95 ± 0.$_{4}$°
PU95CTS5	97 ± 3°	102 ± 1°
PU90CTS10	99 ± 1°	100 ± 2°
PU85CTS15	108 ± 0.$_{3}$°	106 ± 0.$_{2}$°
PU75CTS25	95 ± 0.$_{4}$°	99 ± 0.$_{5}$°
PU50CTS50	96 ± 1°	97 ± 2°
PU25CTS75	97 ± 0.$_{2}$°	100 ± 0.$_{2}$°

Table S5. Values obtained in cytotoxicity assay of PUCTS samples

SYSTEM	Bleached area	Well 1				Well 2				Well 3			
**Control (+)**	quadrant	Q1	Q2	Q3	Q4	Q1	Q2	Q3	Q4	Q1	Q2	Q3	Q4
	extension (em mm)	1.1	1.2	1.0	1.2	1.2	1.5	1.2	1.0	1.0	1.4	1.3	1.3
	values average	1.1				1.2				1.2			
	final average	1.2											
**Control (-)**	quadrant	Q1	Q2	Q3	Q4	Q1	Q2	Q3	Q4	Q1	Q2	Q3	Q4
	extension (em mm)	0	0	0	0	0	0	0	0	0	0	0	0
	values average	0				0				0			
	final average	0											
**PU**	quadrant	Q1	Q2	Q3	Q4	Q1	Q2	Q3	Q4	Q1	Q2	Q3	Q4
	extension (em mm)	0.3	0.3	0.3	0.3	0,3	0,3	0,3	0,3	0,3	0,3	0,3	0,3
	values average	0.3				0.3				0.3			
	final average	0.3											
**PU95CTS5**	quadrant	Q1	Q2	Q3	Q4	Q1	Q2	Q3	Q4	Q1	Q2	Q3	Q4
	extension (em mm)	0.4	0.4	0.4	0.4	0.4	0.4	0.3	0.4	0.4	0.4	0.4	0,4
	values average	0.4				0.4				0.4			
	final average	0.4											
**PU90CTS10**	quadrant	Q1	Q2	Q3	Q4	Q1	Q2	Q3	Q4	Q1	Q2	Q3	Q4

	extension (em mm)	0.2	0.2	0.2	0.2	0.2	0.2	0.2	0.3	0.3	0.2	0.2	0.2
	values average	0.2				0.2				0.2			
	final average	0.2											
**PU85CTS15**	quadrant	Q1	Q2	Q3	Q4	Q1	Q2	Q3	Q4	Q1	Q2	Q3	Q4
	extension (em mm)	0.3	0.2	0.3	0.4	0.2	0.3	0.3	0.3	0.2	0.2	0.3	0.3
	values average	0.3				0.3				0.2			
	final average	0.3											
**PU75CTS25**	quadrant	Q1	Q2	Q3	Q4	Q1	Q2	Q3	Q4	Q1	Q2	Q3	Q4
	extension (em mm)	0.3	0.4	0.4	0.4	0.4	0.4	0.4	0.3	0.3	0.3	0.4	0.3
	values average	0.4				0.4				0.3			
	final average	0.4											
**PU50CTS50**	quadrant	Q1	Q2	Q3	Q4	Q1	Q2	Q3	Q4	Q1	Q2	Q3	Q4
	extensão (em mm)	0.4	0.4	0.3	0.3	0.3	0.3	0.4	0.3	0.3	0.3	0.3	0.3
	values average	0.3				0.3				0.3			
	final average	0.3											
**PU25CTS75**	quadrant	Q1	Q2	Q3	Q4	Q1	Q2	Q3	Q4	Q1	Q2	Q3	Q4
	extension (em mm)	0.3	0.4	0.3	0.4	0.4	0.4	0.3	0.4	0.3	0.3	0.4	0.4
	values average	0.3				0.4				0.3			
	final average	0,3											
